# Clonal transmission of polymyxin B-resistant hypervirulent *Klebsiella pneumoniae* isolates coharboring *bla*_NDM-1_ and *bla*_KPC-2_ in a tertiary hospital in China

**DOI:** 10.1186/s12866-023-02808-x

**Published:** 2023-03-07

**Authors:** Mengli Tang, Jun Li, Zhaojun Liu, Fengjun Xia, Changhang Min, Yongmei Hu, Haichen Wang, Mingxiang Zou

**Affiliations:** 1 Department of Clinical Laboratory, Xiangya Hospital, Central South University, No.87 Xiangya Road, Kaifu district, Changsha, Hunan China; 2 National Clinical Research Center for Geriatric Disorders, Xiangya Hospital, No.87 Xiangya Road, Kaifu District, Changsha, Hunan China

**Keywords:** *Klebsiella pneumoniae*, Polymyxin B, ST11-K64, Hypervirulence, Clonal transmission, Whole-genome sequencing

## Abstract

**Background:**

The prevalence of multidrug-resistant hypervirulent *K. pneumoniae* (MDR-hvKP) has gradually increased. It poses a severe threat to human health. However, polymyxin-resistant hvKP is rare. Here, we collected eight polymyxin B-resistant *K. pneumoniae* isolates from a Chinese teaching hospital as a suspected outbreak.

**Results:**

The minimum inhibitory concentrations (MICs) were determined by the broth microdilution method. HvKP was identified by detecting virulence-related genes and using a *Galleria mellonella* infection model. Their resistance to serum, growth, biofilm formation, and plasmid conjugation were analyzed in this study. Molecular characteristics were analyzed using whole-genome sequencing (WGS) and mutations of chromosome-mediated two-component systems *pmrAB* and *phoPQ*, and the negative *phoPQ* regulator *mgrB* to cause polymyxin B (PB) resistance were screened. All isolates were resistant to polymyxin B and sensitive to tigecycline; four were resistant to ceftazidime/avibactam. Except for KP16 (a newly discovered ST5254), all were of the K64 capsular serotype and belonged to ST11. Four strains co-harbored *bla*_KPC-2_, *bla*_NDM-1_, and the virulence-related genes _*p*_*rmpA*, _*p*_*rmpA2*, *iucA*, and *peg344*, and were confirmed to be hypervirulent by the *G. mellonella* infection model. According to WGS analysis, three hvKP strains showed evidence of clonal transmission (8–20 single nucleotide polymorphisms) and had a highly transferable pKOX_NDM1-like plasmid. KP25 had multiple plasmids carrying *bla*_KPC-2_, *bla*_NDM-1_, *bla*_SHV-12_, *bla*_LAP-2_, *tet(A)*, *fosA5,* and a pLVPK-like virulence plasmid. Tn*1722* and multiple additional insert sequence-mediated transpositions were observed. Mutations in chromosomal genes *phoQ* and *pmrB*, and insertion mutations in *mgrB* were major causes of PB resistance.

**Conclusions:**

Polymyxin-resistant hvKP has become an essential new superbug prevalent in China, posing a serious challenge to public health. Its epidemic transmission characteristics and mechanisms of resistance and virulence deserve attention.

**Supplementary Information:**

The online version contains supplementary material available at 10.1186/s12866-023-02808-x.

## Background

*Klebsiella pneumoniae* (KP) is a common clinical opportunistic pathogen causing various infections in immunocompromised hosts [1]. According to the China Antimicrobial Surveillance Network (https://www.chinets.com/), in 2021, *K. pneumoniae* ranked the second most prevalent isolate (14.12%). Carbapenems are essential antibiotics to treat severe *K. pneumoniae* infections. However, the resistance rates of *K. pneumoniae* to meropenem and imipenem have significantly increased from 2.9% and 3% in 2005 to 26.3% and 25% in 2018 [2]. The global spread of carbapenemases such as *Klebsiella pneumoniae* carbapenemase-2 (KPC-2) and New Delhi metallo-β-lactamase-1 (NDM-1) has exacerbated the health hazards worldwide [3, 4]. With the emergence of carbapenem-resistant isolates, polymyxins have been used in clinical treatment. Polymyxins, mainly including polymyxin B (PB) and polymyxin E (also called colistin), are a group of cationic, basic peptides produced by *Bacillus polymyxa* and have broad-spectrum antibacterial activity against Gram-negative bacteria [5]. Although polymyxins were disfavored due to their potential nephrotoxicity, they are currently used in hospitals and are known as the last line of defense for the clinical treatment of multidrug-resistant Gram-negative bacterial infections. However, as treatment with polymyxins is becoming more common, reports of polymyxin resistance (including chromosomal and plasmid-mediated resistance) are increasing, especially in areas with high polymyxin use [6, 7].

Hypervirulent *Klebsiella pneumoniae* (hvKP) is a new variety of *K. pneumoniae* that can cause severe infections to immunocompetent hosts. According to previous studies, hvKP is usually sensitive to most antibiotics, including cephalosporins and carbapenems [8]. However, recent years the emergence of multidrug-resistant hvKP (MDR-hvKP) has been seen, and the latest research has reported the increasing emergence of colistin-resistant hvKP [9]. Such multidrug-resistant and hypervirulent strains have brought great challenges to clinical anti-infective treatment.

Polymyxin B-resistant hvKP (PBR-hvKP) was rarely reported in previous research. Therefore, to identify the characteristics of PBR-hvKP and to explore its mechanisms of multidrug resistance and hypervirulence, we collected PB-resistant *K. pneumoniae* isolates and conducted in-depth investigations of their clinical and molecular characteristics, including information about their resistance and virulence.

## Results

### Clinical characteristics of eight patients infected with Polymyxin B-resistant *K. pneumoniae*

A total of eight non-repetitive clinical isolates of PB-resistant *K. pneumoniae* were collected. Clinical characteristics of patients infected with PB-resistant *K. pneumoniae* are summarised in Table 1. Six (75%) patients were male, with a mean age of 58.3 ± 15.0 years. The patients came from six different wards, and three (infected with KP17, KP21, and KP25) came from the same intensive care unit (ICU). Main underlying conditions included pneumonia (87.5%, 7/8), hypertension (37.5%, 3/8), abnormal liver function (37.5%, 3/8), tumor (37.5%, 3/8), and cerebral haemorrhage (25%, 2/8). Three patients had a disturbance of consciousness or were in a coma at admission. All the patients had prior antibiotic exposure before detecting PB-resistant *K. pneumoniae*. All the patients underwent invasive procedures, and six patients were admitted to ICU. The main antibiotics used within 72 h after infection included TZP (37.5%, 3/8), MEM (37.5%, 3/8), and TGC (25%, 2/8). Five patients were discharged without recovery. None of the patients died.

**Table 1:** Clinical characteristics of patients infected with PB-resistant *K. pneumoniae*

**Patient**	**Strain**	**Residence time**	**Age/sex**	**Department**	**Underlying conditions**	**ICU stay time**	**Prior antibiotic exposure**	**Source of infection**	**Treated antibiotics in 72 h**	**Discharge status**
1	KP25	2020.11.4–2020.12.20	65/Male	ICU	Multiple fractures, Severe pneumonia, Acute renal injury, Hypertension	47	SCF, LZD, TGC, TZP	Wound	TGC, TZP	Unhealed
2	KP14	2020.11.17–2020.12.4	57/Female	Recovery unit	Pneumonia, Rupture of intracranial aneurysm, Abnormal liver function	8	TZP	Respiratory tract	TZP	critically ill
3	KP17	2021.12.14–2021.2.20	70/Male	ICU	Severe pneumonia, Lung cancer surgery, Hypertension	47	TZP, MEM, PB, VA, CAZ	Respiratory tract	SCF	Improved
4	KP16	2021.1.5–2021.1.15	37/Male	Pancreato-biliary surgery unit	Acute severe pancreatitis	None	CDZ	Digestive tract	CDZ	Improved
5	KP18	2021.1.13–2021.3.15	38/Male	ICU, Neurosurgery unit	Pneumonia, Intracranial infection, Abnormal liver function	71	MEM, PB	Respiratory tract	MEM, TZP	critically ill
6	KP21	2021.2.18–2021.3.26	77/Female	ICU	Hepatocellular carcinoma, Pneumonia, Hepatitis B	8	LVX, CN, SCF	Liver	MEM	Unhealed
7	KP20	2021.3.8–2021.3.19	70/Male	ICU, Respiratory unit	Lymphoma, Severe pneumonia, Abnormal liver function, Type II diabetes	14	MEM, PB	Respiratory tract	MEM, PB	Unhealed
8	KP24	2021.3.25–2021.5.27	52/Male	Integrated traditional Chinese and western medicine unit	Brainstem hemorrhage, Pneumonia, Hypertension, Type II diabetes	None	PB, TGC	Urinary tract	TGC	Improved

Abbreviations: *ICU* Intensive care unit, *SCF* Cefoperazone/sulbactam, *LZD* Linezolid, *TGC* Tigecycline, *TZP* Piperacillin/tazobactam, *MEM* Meropenem, *PB* Polymyxin B, *VA* Vancomycin, *CDZ* Cefodizime, *LVX* Levofloxacin, *CN* Gentamicin

### Antimicrobial susceptibility and antibiotic resistance genes

**Fig. 1:**
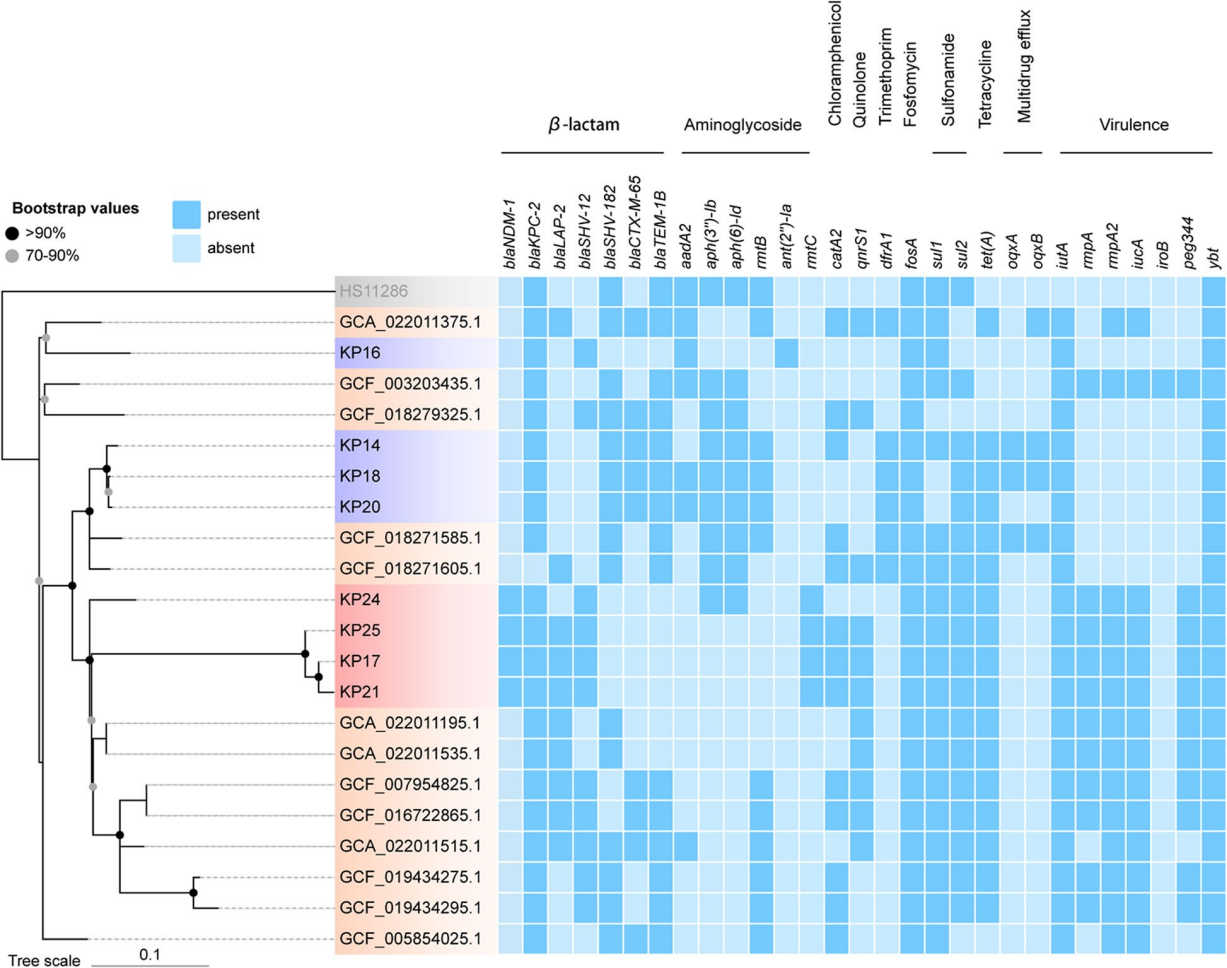
Phylogenetic relationship, resistance genes, and virulence genes of 8 PB-resistant strains (4 PBR-hvKP strains with red background, 4 PB-resistant strains with purple background) in this study and 13 ST11 PBR-hvKP strains from other provinces in China. The darker blue box represents the presence of genes. *K. pneumoniae* HS11286 was used as the reference for core SNPs calling

All isolates were resistant to PB and sensitive to TGC; four were resistant to CZA (Table 2). All eight PB-resistant isolates carried carbapenemase gene *bla*_KPC-2_ and fosfomycin-resistance gene *fosA*; four isolates carried carbapenemase gene *bla*_NDM-1_; five isolates carried extended-spectrum β-lactamase gene *bla*_SHV-12_; and three isolates carried *bla*_SHV-182_, *bla*_CTX-M-65_, and *bla*_TEM-1B_ (Fig. 1). The plasmid-borne colistin resistance gene *mcr-1* was not detected. All isolates were found to have D150G amino acid substitutions in the *phoQ* gene and R256G amino acid substitution in the *pmrB* gene. Four isolates had different amino acid substitutions in the *pmrB* gene, including T157P and S85R. IS*Kpn74*-like and IS*903B*-like insertion mutations in the *mgrB* gene were detected in two isolates (Table 2).

**Table 2:** Antimicrobial susceptibility results of PB-resistant *K. pneumoniae* and the *E. coli* transconjugants and substitution mutations of chromosomal regulators in PB-resistant isolates

		Minimum inhibitory concentrations (μg/mL)												Mutations of chromosomal regulators		
Strain	**Category**	**CAZ**	**FEP**	**CZA**	**ATM**	**TZP**	**NIT**	**IPM**	**MEM**	**AMK**	**LVX**	**TGC**	**PB**	***mgrB***	***phoQ***	***pmrB***
KP14	XDR	**64**	**> 128**	≤ 1/4	**> 128**	**> 512/4**	**512**	**> 32**	**> 32**	**> 512**	**> 16**	1	**> 16**	**-**	D150G	R256G
KP16	MDR	**> 128**	**> 128**	≤ 1/4	**> 128**	**> 512/4**	**256**	**32**	**32**	≤ 4	**16**	0.5	**16**	**-**	D150G	R256G
KP17	XDR	**> 128**	**> 128**	**> 128/4**	**> 128**	**> 512/4**	**256**	**> 32**	**> 32**	**> 512**	**> 16**	0.5	**16**	**-**	D150G	T157P, R256G
KP18	XDR	**64**	**> 128**	≤ 1/4	**> 128**	**> 512/4**	**512**	**> 32**	**> 32**	**> 512**	**> 16**	1	**> 16**	IS*Kpn74*	D150G	R256G
KP20	XDR	**128**	**> 128**	1	**> 128**	**> 512/4**	**256**	**> 32**	**> 32**	**> 512**	**16**	0.5	**16**	**-**	D150G	S85R, R256G
KP21	XDR	**> 128**	**> 128**	**> 128/4**	**> 128**	**> 512/4**	**128**	**> 32**	**> 32**	**> 512**	**> 16**	1	**16**	**-**	D150G	T157P, R256G
KP24	XDR	**> 128**	**> 128**	**> 128/4**	**> 128**	**> 512/4**	**512**	**> 32**	**> 32**	**> 512**	**> 16**	2	**> 16**	IS*903B*	D150G	R256G
KP25	XDR	**> 128**	**> 128**	**> 128/4**	**> 128**	**> 512/4**	**256**	**> 32**	**> 32**	**> 512**	**16**	0.5	**16**	**-**	D150G	T157P, R256G
EC600	-	≤ 1	≤ 1	≤ 1/4	≤ 1	8/4	8	≤ 0.25	≤ 0.25	≤ 4	≤ 0.125	≤ 0.125	0.5	-	-	-
KP17-T	MDR	**> 128**	**64**	**> 128/4**	≤ 1	**256/4**	8	**16**	**16**	**> 512**	≤ 0.125	≤ 0.125	0.5	-	-	-
KP21-T	MDR	**> 128**	**64**	**> 128/4**	≤ 1	**512/4**	8	**8**	**16**	**> 512**	0.25	≤ 0.125	0.5	-	-	-
KP25-T	MDR	**> 128**	**32**	**> 128/4**	≤ 1	**256/4**	8	**16**	**16**	**> 512**	≤ 0.125	≤ 0.125	0.5	-	-	-

KP17-T, KP21-T, KP25-T, Transconjugants

Abbreviations: *CAZ* Ceftazidime, *FEP* Cefepime, *CZA* Ceftazidime/avibactam, *ATM* Aztreonam, *TZP* Piperacillin/tazobactam, *NIT* Nitrofurantoin, *IPM* Imipenem, *MEM* Meropenem, *AMK* Amikacin, *LVX* Levofloxacin, *TGC* Tigecycline, *PB* Polymyxin B

### Virulence phenotype and genetic characteristics

**Fig. 2:**
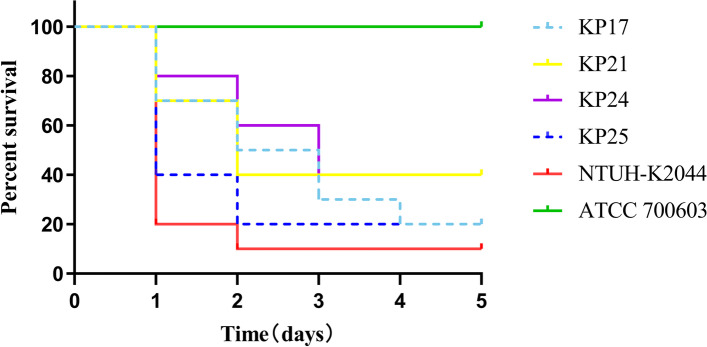
Four PBR-hvKP infections of *G. mellonella* larvae. The survival rate of larvae infected with KP17, KP21, KP24, and KP25 was 20.0%, 40.0%, 40.0%, and 20.0%. NTUH-K2044 and ATCC 700603 were used as high and low virulence control strains

**Fig. 3:**
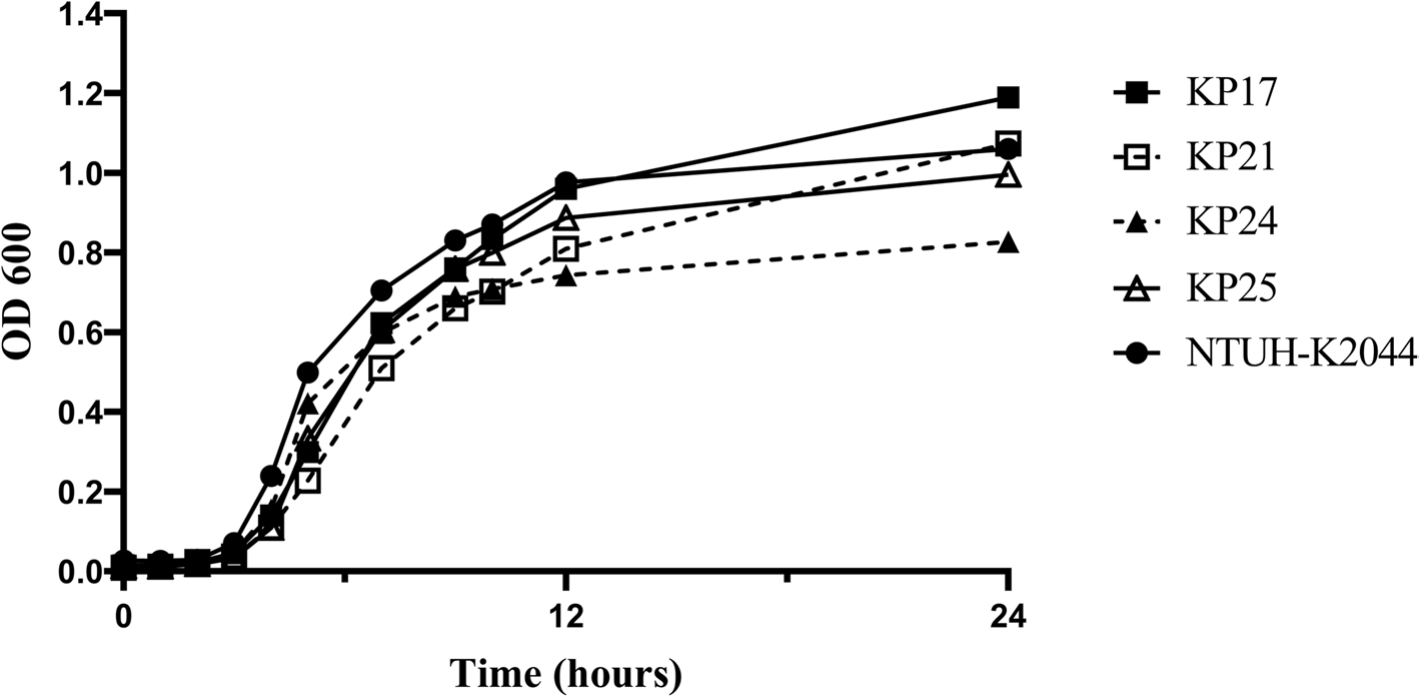
Growth curves of four PBR-hvKP. NTUH-K2044 was used as a high virulence control strain

All isolates were non-mucoviscous according to the string test. Four isolates (KP17, KP21, KP24, and KP25) carried hypervirulent biomarker genes *rmpA*, *rmpA2*, *peg344*, and *iucA* (Fig. 1). It was worth noting that KP17, KP21, and KP25 had in-frame truncations in *rmpA2*, which resulted in a 636-bp sequence variation. Results of the *G. mellonella* infection model showed that the five-day mortality rates of these isolates were greater than or equal to 60% (Fig. 2), suggesting that they were PBR-hvKP. Except for KP24, almost all isolates could form biofilms. Six were classified as strong film-forming isolates, and one was classified as a moderate film-forming isolate. All PBR-hvKP isolates were sensitive to serum. However, the growth abilities of four PBR-hvKP differed (Fig. 3). After 8–12 h of monitoring, the growth of KP17 was the strongest, while the growth of KP24 was weak (*P* < 0.05).

### MLST genotyping, serotypes, and phylogenetic analysis

Features of genomes of eight PB-resistant *K. pneumoniae* isolates are included in Supplementary Table S1. Table 3 shows the origin, types, major resistance and virulence characteristics, capsular serotypes, and sequence types (STs) of eight PB-resistant isolates. ST11 was the predominant ST (7/8, 87.5%), except for KP16, which belonged to ST5254. ST5254 is a newly discovered ST type, a single-locus (*phoE*) variant of ST11, with the *phoE515* gene in ST5254 differing from *phoE1* in ST11 (Supplementary Table S2). All PB-resistant isolates belonged to the K64 serotype. In order to further clarify the relationship between our PB-resistant strains and those reported in China, we reviewed colistin/PB-resistant hypervirulent ST11 strains reported previously. A total of 13 genomes of colistin/PB-resistant hypervirulent ST11 strains were downloaded from NCBI and used for phylogenetic analysis together with genomes in this study (Fig. 1). The results showed that strains in this study had specific differences in affinity with those reported before and did not belong to the same cluster. Notably, three PBR-hvKP KP17, KP21, and KP25 were classified as the same cluster. The SNPs ranged from 8 to 20 (Supplementary Figure S1), indicating probable clonal transmission [10]. Another cluster included KP14, KP20, and KP18. KP16 and KP24 were individual isolates. The distribution of colistin/PB-resistant hypervirulent ST11 *K. pneumoniae* in those clades indicated that PBR-hvKP KP17, KP21, and KP25 belonged to a new clade with *bla*_NDM-1_.

**Table 3:** Characteristics of eight PB-resistant *K. pneumoniae*

Strains	Patient	Specimen	STs	Capsular type	Strain type	Carbapenem resistance genes	String test	Virulence genes	Biofilm assay	Serum resistance
KP14	2	Sputum	11	K64	XDR-KP	*bla*_KPC-2_	-	-	+ + +	0.73%
KP16	4	Drainage	5254	K64	MDR-KP	*bla*_KPC-2_	-	-	+ +	0.08%
KP17	3	Sputum	11	K64	XDR-hvKP	*bla*_NDM-1_*, bla*_KPC-2_	-	*rmpA*, *rmpA2*, *peg344*, *iucA*	+ + +	0.00%
KP18	5	Sputum	11	K64	XDR-KP	*bla*_KPC-2_	-	-	+ + +	0.62%
KP20	7	Sputum	11	K64	XDR-KP	*bla*_KPC-2_	-	-	+ + +	40.00%
KP21	6	Blood	11	K64	XDR-hvKP	*bla*_NDM-1_*, bla*_KPC-2_	-	*rmpA*, *rmpA2*, *peg344*, *iucA*	+ + +	0.00%
KP24	8	Urine	11	K64	XDR-hvKP	*bla*_NDM-1_*, bla*_KPC-2_	-	*rmpA*, *rmpA2*, *peg344*, *iucA*	-	0.00%
KP25	1	Wound	11	K64	XDR-hvKP	*bla*_NDM-1_*, bla*_KPC-2_	-	*rmpA*, *rmpA2*, *peg344*, *iucA*	+ + +	1.15%

Abbreviations: *XDR-KP* Extensively drug-resistant *K. pneumoniae*, *MDR-KP* Multidrug-resistant *K. pneumoniae*, *XDR-hvKP* Extensively drug-resistant hypervirulent *K. pneumoniae*

### Complete genomic and comparative analysis of KP25

The KP25 genome contained one chromosome with a length of 4,588,001 bp and seven plasmids. Table 4 summarises the genome characteristics of KP25. The chromosome carried type 3 fimbriae gene *mrkABCDFHJ*, and yersiniabactin siderophore genes *ybtPQSX–ybtAUTE*. The major plasmids included ColRNAI (plasmid 2), IncFII (Yp) (plasmid 3), IncFII (pHN7A8) (plasmid 4), IncFII (pCRY) (plasmid 6), and repB (plasmid 7). Drug resistance genes *bla*_NDM-1_ (in plasmid 3), *bla*_KPC-2_ (in plasmid 4), *tet(A)* (in plasmid 6), and virulence genes *rmpA*, *rmpA2*, *iutA*, *iucB*, *iucC*, and *peg344* (in plasmid 7) were distributed in different plasmids.

**Table 4:** Genome characteristics of isolate KP25

Genetic context	Length (bp)	GC content (%)	Plasmid type	Closest plasmid match (identity)	#MEGs	Resistance genes	Virulence genes
Chromosome	4,588,001	57.39	-	-	37	-	*mrkABCDFHJ*, *ybtPQSX–ybtAUTE*
Plasmid 1	743,487	57.38	-	-	3	*fosA5*	-
Plasmid 2	23,917	55.57	ColRNAI	pKP8695-p4 (99.76%)	0	-	-
Plasmid 3	110,782	54.83	IncFII (Yp)	pRJF866 (99.92%)	10	*bla*_NDM-1_, *sul1*, *rmtC*	-
Plasmid 4	92,836	55.21	IncFII (pHN7A8)	p12085-KPC (99.92%)	12	*bla*_KPC-2_, *bla*_SHV-12_	-
Plasmid 5	91,870	56.99	-	-	0	-	-
Plasmid 6	83,552	54.00	IncFII (pCRY)	p16ZR-187-IncFII-83-R (99.99%)	2	*tet(A)*, *catA2*, *sul2*, *qnrS1*, *bla*_LAP-2_	-
Plasmid 7	219,442	49.93	repB	pK2044 (99.29%)	21	-	*rmpA*, *rmpA2*, *iutA*, *iucB*, *iucC, peg344*

#MEGs, the number of mobile genetic elements

**Fig. 4:**
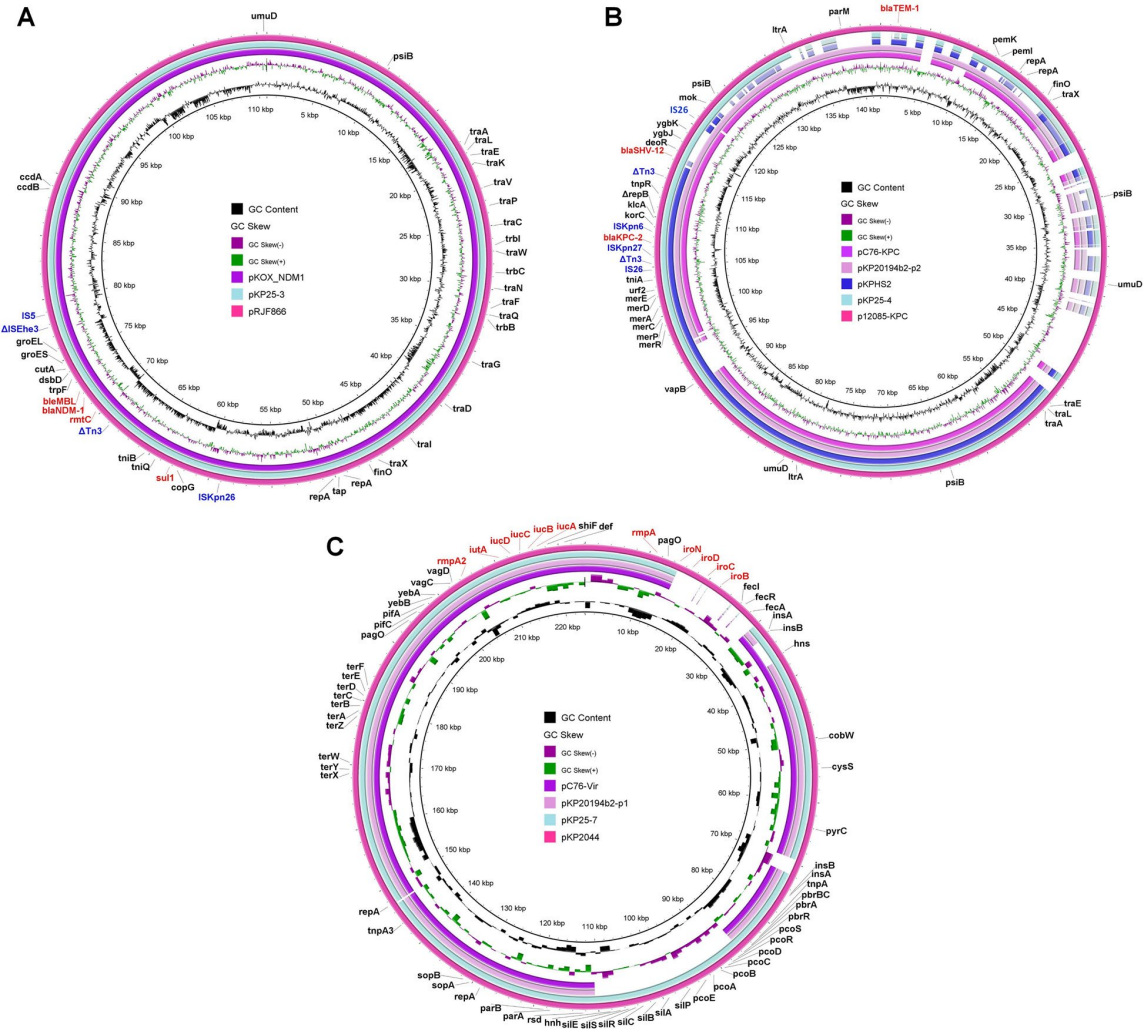
Genetic comparison of *bla*_NDM-1_-carrying plasmid (**A**), *bla*_KPC-2_-carrying plasmid (**B**), and virulence plasmid (**C**) from KP25, including the best matching plasmids; plasmids from KP-C76 [11] and plasmids from KP20194b2 [12]

**Fig. 5:**
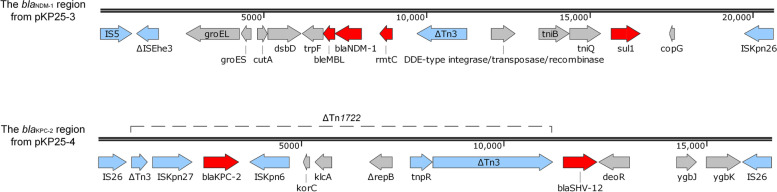
The *bla*_NDM-1_ region from plasmid 3 and the *bla*_KPC-2_ region from plasmid 4 in KP25. Genes are depicted as arrows according to the direction of transcription. Resistance genes are in red, mobile elements are in blue, and other traits are in grey

The most similar genomes to KP25 were previously reported carbapenem-resistant *K. pneumoniae* (CR-KP) strains KP-C76 [11] and KP20194b2 [12] isolated respectively in Hangzhou and Hengyang in China, both containing a *bla*_KPC-2_-carrying plasmid (Fig. 4B), a *tet(A)*-carrying plasmid, and a pLVPK-like virulence plasmid (Fig. 4C). In the *bla*_KPC-2_-carrying plasmid (pKP25-4), the genetic environment of *bla*_KPC-2_ and *bla*_SHV-12_ was IS*26*-ΔTn*3*-IS*Kpn27*-*bla*_KPC-2_-IS*Kpn6*-*korC*-*klcA*-Δ*repB*-*tnpR*-ΔTn*3*-*bla*_SHV-12_-*deoR*-*ygbJ*-*ygbK*-IS*26* (Fig. 5), which was a composite transposon consisting of Tn*1722* in pKPHS2 (GenBank no. CP003224.1) and a truncated IS*26*-*bla*_SHV-12_-IS*26* unit [13]. The *tet(A)*-carrying plasmid (pKP25-6) carried an MDR region consisting of multiple resistance genes *sul2*, *catA2*, *tet(A)*, *bla*_LAP-2_, *qnrS1*, and transposases IS*26*, IS*5075*, IS*Vsa3*, and IS*Kpn19*. β-lactamase gene *bla*_LAP-2_ was flanked upstream by ΔTn*1721* and presumed to exist in a truncated IS*26*-*bla*_LAP-2_-*qnrS1*-IS*26* unit. The virulence plasmid (pKP25-7) had a high similarity (99.29%) with the virulence plasmid pK2044 carried by the reference strain NTUH-K2044. It was worth noting that *rmpA* and *peg344* were flanked upstream by IS*Kpn26* and downstream by IS*903B*. The stop codon TAG in *rmpA2* led to a fragment deletion. Interestingly, the *bla*_NDM-1_-carrying plasmid (pKP25-3) was not present in previous similar genomes of KP-C76 and KP20194b2. It shared 99.92% nucleotide identity with the published plasmid pRJF866 (GenBank no. KF732966) found in a CR-KP isolate from a blood specimen from a hospital in Shanghai (Fig. 4A), which was a pKOX_NDM1-like plasmid [14]. Sequence analysis showed that *bla*_NDM-1_, *sul1*, and *rmtC* formed an MDR region flanked upstream by IS*5* and downstream by IS*Kpn26* (Fig. 5). The genetic environment was IS*5*-ΔIS*Ehe3*-*groEL*-*groES*-*cutA*-*dsbD*-*trpF*-*ble*_MBL_-*bla*_NDM-1_-*rmtC*-ΔTn*3*- DDE-type integrase/transposase/recombinase-*tniB*-*tniQ*-*sul1*-*copG*-IS*Kpn26*.

Conjugation assays showed that the plasmid carrying *bla*_NDM-1_ could be successfully transferred to *E. coli* EC600 from KP17, KP21, and KP25. The transconjugants were resistant to CAZ, FEP, CZA, TZP, IPM, MEM, and AMK (Table 2), whereas the plasmid-carrying *bla*_KPC-2_ was not transferred under experimental conditions.

## Discussion

The resistance rates of hvKP were generally lower than those of classical *K. pneumoniae* (cKP) reported in previous studies [15]. However, recent studies in many countries have reported the emergence of MDR-hvKP. The fatality rate of MDR-hvKP infected patients was 56.3%-66.7% in China, and the rate of isolation of MDR-hvKP strains is increasing [16–18]. Polymyxins are essential antibiotics for Gram-negative bacteria exhibiting MDR. In this study, we collected eight strains of PB-resistant *K. pneumoniae* and analyzed the clinical and molecular characterization of these strains.

**Fig. 6:**
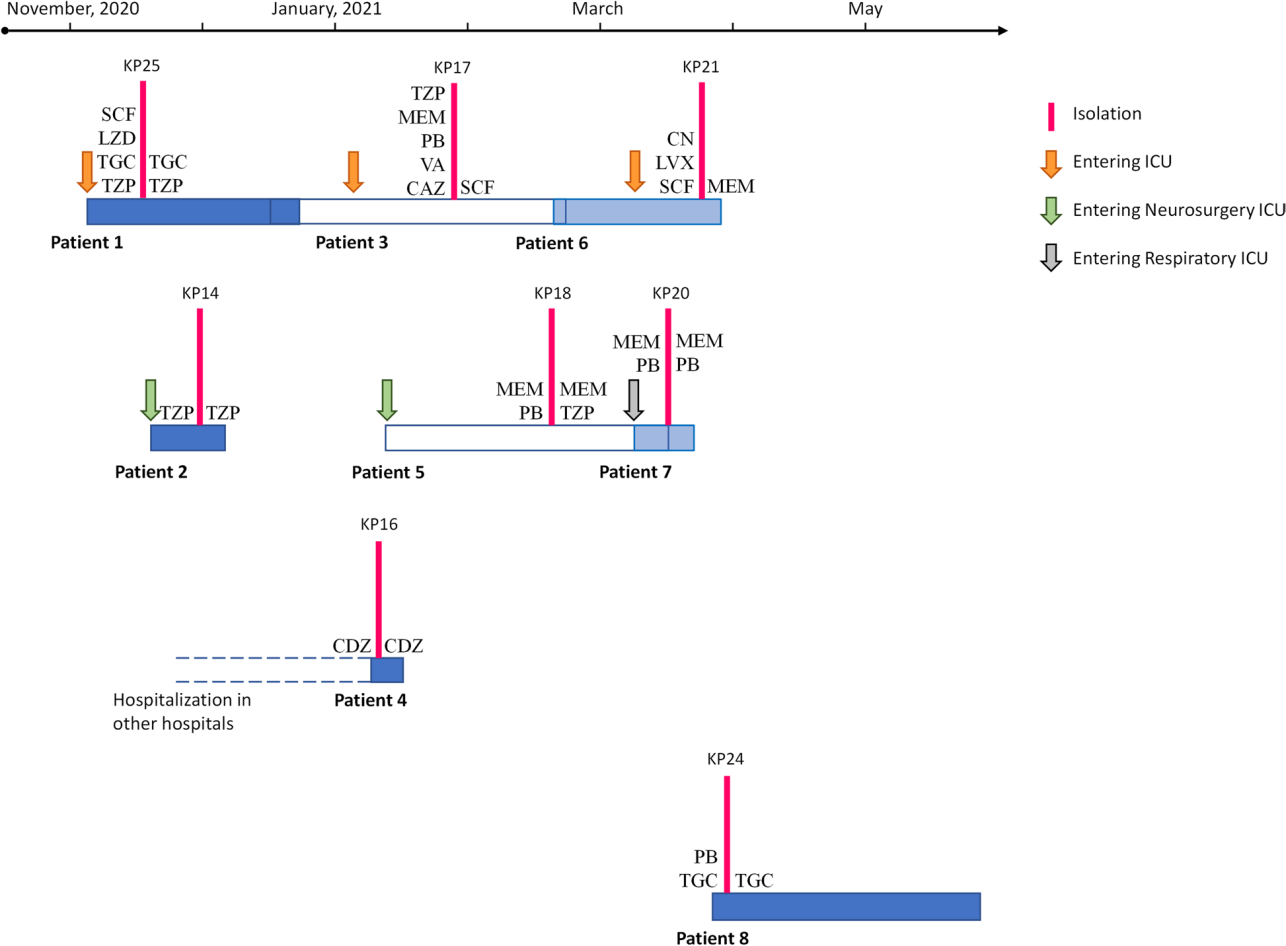
Epidemiology of eight PB-resistant *K. pneumoniae* cases. The rectangles on the timeline represent the duration of the patient’s admission. Antibiotics used before and after the isolation of PB-resistant strains were marked

A clonal transmission of ST11-K64 PB-resistant *K. pneumoniae* in the hospital and two clusters among these isolates were investigated. Epidemiological information showed that patients were admitted to different ICU wards, and patients 1, 3, and 6 had overlapping stays (Fig. 6). Following the fatal outbreak of ST11-K47 CR-KP in ICU wards [19], this study indicated that ST11 PBR-hvKP formed a new clade in China and spread within a hospital setting. Most of the strains isolated in this study were ST11, except KP16, which was first discovered ST5254. ST307, ST512 and ST147 have been shown to be hyperepidemic CR-KP clones in Europe [20–22]. In China, ST11 and ST23 have been the main ST types of MDR-KP, and the KPC-2-producing ST11 was the dominant clone of CR-KP [23]. In the present study, we reported ST5254 for the first time and demonstrated that this new ST is a high-risk clone resistant to PB and carries MDR genes such as *bla*_KPC-2_, which requires close attention. The identification of clinically isolated *K. pneumoniae* by *wzc* or *wzi* gene sequencing has revealed that K64 is one of the major serotypes of CR-KP [24]. ST11-K64 CR-hvKP carried MDR genes such as *bla*_KPC-2_ and *bla*_NDM-1_ together with pLVPK-like virulence plasmid, expressing high virulence in a *G. mellonella* infection model, and the fatality rate of patients was high [12, 25, 26].

All PB-resistant isolates in this study carried at least two common mutations, including D150G in *phoQ* and R256G in *pmrB* [27–29]. Homologous PBR-hvKP strains showed consistent mutation patterns, whereas new mutations were derived in other strains. T157P in *pmrB* caused overexpression of the operons *pmrCAB* and *pmrHFIJKLM*, resulting in the increase of MICs of PB in clinical isolates [30]. S85R in *pmrB* was predicted to affect protein function by PROVEAN (http://provean.jcvi.org/seq_submit.php) (Supplementary Figure S2). Insertion mutation and inactivation of *mgrB* led to overexpression of the *phoPQ* operon, which activated the *pmrHFIJKLM* operon and produced 4-amino-4-deoxy-L-aminoarabinose (L-Ara4N), leading to resistance [31]. Resistance to polymyxins can be influenced by external environmental stimuli such as antibiotics. Choi et al. [32] showed that under the stimulation of colistin, mutations in *phoP*, *phoQ*, and *pmrB* were generated in PB-sensitive ST23-KP. The acquisition of drug resistance occurs at the cost of reducing capsular polysaccharides and virulence. Murtha et al. [33] studied Enterobacterales and found that meropenem could induce PhoPQ and increase modifications of L-Ara4N. Exposure to different antibiotics can lead to changes in bacterial tolerance. Long-term hospitalization and the use of β-lactams increase the risk of PB-resistant infections.

WGS and comparative analysis revealed the molecular characterization of PB-resistant MDR-hvKP. PBR-hvKP strains in this study were related to isolates previously reported in Hangzhou (KP-C76) and Hengyang (KP20194b2), China [11, 12]. Similarly, KP-C76 was also found to develop colistin resistance due to the insertion mutation of *mgrB*. However, it differed from mutations in KP25, suggesting that the resistant mutations of polymyxins were relatively independent in different strains. Another difference was that KP25 obtained a pKOX_NDM1-like plasmid, which was firstly discovered in Taiwan and spread in eastern China, such as Shanghai and Jiangxi [14, 34, 35]. The plasmid carrying *bla*_NDM-1_ made PBR-hvKP resistant to CZA. Class B metallo-β-lactamase and mutations in *bla*_KPC_ are the main mechanisms of CZA resistance [36, 37]. Conjugation assays showed that the pKOX_NDM1-like plasmid demonstrated a stronger transfer ability than the *bla*_KPC-2_-carrying plasmid [14, 38]. pKP25-3 carries the entire *tra* region (Fig. 4A), which encodes the highly conserved gene products of the type IV secretion system, while in pKP25-4, the region was inserted by IS*26* and led to gene deletion (Fig. 4B), which explains the low conjunction rate of the *bla*_KPC-2_ plasmid [39]. The conjugation and expression of the plasmid carrying *bla*_NDM-1_ and *rmtC* endowed *E. coli* EC600 with resistance to CAZ, FEP, CZA, TZP, IPM, MEM, and AMK, while the chromosome-mediated resistant genes and *qnrS1*-carrying plasmid were not conjugated. Strains co-harboring *bla*_KPC-2_ and *bla*_NDM-1_ were able to spread stably in the environment. The *bla*_NDM-1_ gene is highly transferable between different species in Enterobacterales, expanding the drug resistance spectrum [38].

Virulence-related genes *mrk* coding for type 3 fimbriae and *ybt* coding for yersiniabactin in the chromosome, aerobactin genes *iuc*/*iutA* and salmochelin gene *iro* in pLVPK-like virulence plasmid were found in PBR-hvKP [40]. Four PBR-hvKP isolates in this study carried *rmpA* and *rmpA2*. However, the *rmpA2* was a truncated 636-bp fragment with a frameshift mutation, which may be responsible for the loss of mucus phenotype and weak serum resistance [41]. In fact, frameshift *rmpA2* was not uncommon in ST11-K64 isolates. Zhou et al. [42] reported the presence of frameshift mutations in 52.2% (48/92) *rmpA2*-positive ST11-K64 isolates. The virulence of strains with mutant *rmpA2* was not significantly different from that of wild strains in the *G. mellonella* infection model. However, the environmental survival rate was significantly increased and had the advantage of hospital transmissibility. On the other hand, loss of hypermucoviscosity and weak serum resistance may be associated with the acquisition of PB resistance [32]. Stimulation of PB led to enhanced resistance in mutant strains, accompanied by the reduction in virulence-related phenotypes. Strong film-forming ability and low mucus phenotype increased the ability of strains to colonize and spread in hospitals, leading to persistent chronic infections in patients [43].

## Conclusions

In conclusion, we studied the clonal transmission and molecular characterization of co-harboring *bla*_NDM-1_ and *bla*_KPC-2_ PBR-hvKP in a tertiary hospital in China. PBR-hvKP not only carried multiple resistance genes but also had hypervirulence. Mutations in chromosomal genes *phoQ* and *pmrB*, and insertion mutations in *mgrB* were major causes of PB resistance. In addition, we discovered a novel ST type, ST5254, which broadened the types of pan-resistant *K. pneumoniae*. ST11-K64 is a new type of PBR-hvKP epidemic in China. Rapid remodeling and diversification of genomes and promoting mobile genetic elements make it a new crucial super bacterium. Therefore, strengthening the clinical monitoring of ST11-K64 strains and taking effective measures to avoid transmission of resistant bacteria is significant for preventing and treating MDR-hvKP infections.

## Methods

### Polymyxin B-resistant *K. pneumoniae* strains and data collection

We conducted a retrospective study at Xiangya Hospital, Central South University. *K. pneumoniae* isolates were collected from clinical specimens processed routinely between November 2020 and April 2021; these isolates were chosen based on being resistant to PB as involved in a suspected outbreak. Only the first culture was included for patients with two or more PB-resistant *K. pneumoniae* strains. Matrix-assisted laser desorption/ionization time-of-flight mass spectrometry (MALDI-TOF MS; Bruker Daltonics GmbH, Bremen, Germany) and the Vitek 2 Compact System (BioMérieux, Marcy l'Etoile, France) were used for identification and antimicrobial susceptibility testing. In addition, patients' clinical data were collected from hospital computer databases.

### Antimicrobial susceptibility test

Antimicrobial susceptibility testing was performed using the broth microdilution method. The antibiotics (Wenzhou Kangtai Biotechnology Company, Wenzhou, China) included ceftazidime (CAZ), cefepime (FEP), ceftazidime/avibactam (CZA), aztreonam (ATM), piperacillin/tazobactam (TZP), nitrofurantoin (NIT), imipenem (IPM), meropenem (MEM), amikacin (AMK), levofloxacin (LVX), tigecycline (TGC), and PB. The minimum inhibitory concentrations (MICs) of the antibiotics, except that of TGC, were in accordance with those defined by the Clinical and Laboratory Standards Institute (CLSI) 2021 breakpoints [44]. Susceptibility to TGC was interpreted according to the US Food and Drug Administration (US-FDA) breakpoints (https://www.fda.gov/drugs/development-resources/tigecycline-injection-products). *E. coli* ATCC 25922 was used for quality control.

### Detection of capsular type, hypermucoviscosity, and virulence-related genes _*p*_*rmpA*, _*p*_*rmpA2*, *iroB*, *peg344*, and *iucA*

Capsular type of *K. pneumoniae* was detected by polymerase chain reaction (PCR), and the *wzi* locus was sequenced as previously described [45]. Mucoviscous phenotype was evaluated using the string test [46]. Isolates were grown on blood agar plates overnight at 37 °C, and colonies were gently touched and lifted with an inoculation loop. Hypermucoviscosity was defined as a macroscopically observed mucus string > 5 mm in length. Virulence-related genes plasmid-borne *rmpA* (_*p*_*rmpA*), _*p*_*rmpA2*, *iroB*, *peg344*, and *iucA* were detected by PCR to identify hypervirulent strains as previously [44]. In short, DNA was extracted by boiling method and specific primers, and PCR conditions were according to the previous study [47]. PCR was performed using an Applied Biosystems thermal cycler (Applied Biosystems, Foster City, CA) as follows: denaturation for 5 min at 94 °C, 30 cycles of 30 s at 95 °C, 30 s at primer-specific annealing temperature, and 30 s at 72 °C, and a final extension for 10 min at 72 °C.

### *Galleria mellonella* infection model

The virulence of the strain was evaluated using *G. mellonella* larvae (Tianjin Huiyude Biotechnology Company, Tianjin, China). The experiment was modified as described in a previous study [48]. First, the strains to be tested were cultured overnight; then, a single colony was selected and used to prepare 1.0 × 10^7^ colony forming units (CFU)/mL bacterial solution with phosphate-buffered saline (PBS). Each larva was injected with 10 μL of the bacterial suspension through the last left proleg, then placed in disposable plates and incubated at 37 °C in the dark for 5 d. The number of deaths was recorded every 24 h. NTUH-K2044 and *K. pneumoniae* ATCC 700603 (National Clinical Laboratory Center, Beijing, China) were used as high- and low-virulence control strains, respectively. Tests were performed in triplicate.

### Serum resistance assay

The serum resistance assay was conducted as previously described [49]. Strains were cultured in Luria–Bertani (LB) medium overnight, then washed and suspended with PBS. Then, 100 μL of bacterial suspension was added to 300 μL of healthy human serum and incubated at 37 °C for 3 h. Before and after co-cultivation, 100 μL of suspension was taken to determine the CFUs. The bacterial killing effect was defined as the percentage of CFU after culture in serum compared with the bacterial CFU before culture. Tests were performed in triplicate.

### Growth curves and biofilm assay

The growth curves were made following a previous study [50]. First, a single colony of bacteria was standardized to match a 0.5 McFarland followed by 1:100 dilution in LB broth at 37 °C with medium shaking. Then, 200 μL of the bacterial solution was taken out, and the optical density at 600 nm (OD_600_) was measured every hour during the first 12 h. Finally, multiple *t*-test was used to compare growth at various time points.

Biofilm formation was assessed by the crystal violet staining method [51]. The strains were prepared into 1.0 × 10^8^ CFU/mL solution with PBS, diluted to 1:100 with LB broth, and cultured in a 96-well plate (3 parallel wells for each strain) at 37 °C for 24 h. The plate was then washed with PBS, stained with 1% crystal violet for 15 min, and then decolorized with 95% ethanol for 10 min. Absorbance was measured at OD_570_. The classification standard followed the previous study [52]. Growth curves and biofilm assay were performed in triplicate.

### Whole-genome sequencing (WGS) and data analysis

Genomic DNA was isolated using the MagAttract HMW DNA Kit (Qiagen, Hilden, Germany) and submitted to next-generation high-throughput sequencing (NGS) on a HiSeq 2000 platform (Illumina Inc., San Diego, CA, USA) with 2 × 100-bp paired-end reads. Pacbio sequel II and DNBSEQ platform (Beijing Genomics Institute, Shenzhen, China) were used for sequencing of the Genomic DNA of KP25. The Canu program was used for self-correction. GATK (https://www.broadinstitute.org/gatk/) was used to make single-base corrections. The whole-genome sequence was annotated by the RAST tool version 2.0 (https://rast.nmpdr.org/). The multilocus sequence typing (MLST) profiles were determined with the MLST database (https://bigsdb.pasteur.fr/klebsiella/). Snippy was applied to run core single-nucleotide polymorphisms (SNPs) calling (https://github.com/tseemann/snippy) and generate a phylogenetic tree based on the maximum-likelihood method with *K. pneumoniae* HS11286 (GenBank no. CP003200.1) as the reference. ChiPlot (https://www.chiplot.online/) was used for the visualization of the phylogenetic tree. The antibiotic resistance genes and virulence loci of the assembled genome sequences were identified using ResFinder 4.1 (https://cge.cbs.dtu.dk/services/ResFinder/) and the MLST database. PlasmidFinder 2.1 (https://cge.cbs.dtu.dk/services/PlasmidFinder/) was used to predict the plasmid types. ISfinder database (https://www-is.biotoul.fr/index.php) was used to determine the insert sequences. A comparison of sequences of plasmids was conducted using BRIG [53]. The sequences of PB-resistant strains are available in the National Center for Biotechnology Information (NCBI) database under the BioProject accession number PRJNA824787.

### Bacterial conjugation

PB-resistant strains were used as the donors (meropenem resistant), and *E. coli* EC600 (rifampicin resistant) was used as the recipient strain for plasmid-binding assays. The donor and recipient strains cultured in LB broth overnight were mixed and spotted on sterile filter paper, then incubated on a blood plate at 37 °C for 18 h. Transconjugants were selected on MacConkey agar supplemented with 1 mg/L meropenem and 600 mg/L rifampicin. *bla*_NDM-1_ PCR and antimicrobial susceptibility test of meropenem were performed to confirm the transconjugants.

## Supplementary Information

**Additional file 1:** **Table S1.** Features of genomes of eight PB-resistant *K. pneumoniae* isolates. **Table S2.** Allelic profiles of eight PB-resistant *K. pneumoniae* isolates. **Figure S1.** Paired SNP distance of eight PB-resistant *K. pneumoniae* isolates. **Figure S2.** PROVEAN result shows the effect of S85R in PmrB.

## Data Availability

The data supporting this study's findings are openly available in the NCBI BioProject repository at https://www.ncbi.nlm.nih.gov/bioproject/824787 under BioProject accession number PRJNA824787.

## Abbreviations

MDR: Multidrug-resistant
hvKP: Hypervirulent *K. pneumoniae*
MICs: Minimum inhibitory concentrations
WGS: Whole-genome sequencing
KP: *Klebsiella* *pneumoniae*
KPC-2: *Klebsiella** pneumoniae* carbapenemase-2
NDM-1: New Delhi metallo-β-lactamase-1
PB: Polymyxin B
PBR-hvKP: Polymyxin B-resistant hypervirulent *K. pneumoniae*
MALDI-TOF MS: Matrix-assisted laser desorption/ionisation time-of-flight mass spectrometry
CAZ: Ceftazidime
FEP: Cefepime
CZA: Ceftazidime/avibactam
ATM: Aztreonam
TZP: Piperacillin/tazobactam
NIT: Nitrofurantoin
IPM: Imipenem
MEM: Meropenem
AMK: Amikacin
LVX: Levofloxacin
TGC: Tigecycline
CLSI: Clinical and Laboratory Standards Institute
US-FDA: US Food and Drug Administration
PCR: Polymerase chain reaction
CFU: Colony forming units
PBS: Phosphate-buffered saline
LB: Luria–Bertani
cgMLST: Core genome multilocus sequence typing
MLST: Multilocus sequence typing
NCBI: National Center for Biotechnology Information
ICU: Intensive care unit
CR-KP: Carbapenem-resistant *K. pneumoniae*
cKP: Classical *K. pneumoniae*
XDR: Extensively drug-resistant
L-Ara4N: 4-Amino-4-deoxy-L-aminoarabinose
