# Supplementary material for: Clonal transmission of polymyxin B-resistant hypervirulent Klebsiella pneumoniae isolates coharboring blaNDM-1 and blaKPC-2 in a tertiary hospital in China
Source: BMC Microbiol. 2023 Mar 7;23:64. doi: 10.1186/s12866-023-02808-x (PMC9990273; doi:10.1186/s12866-023-02808-x)
Supplement: Supplementary file 1 — Additional file 1: Table S1. Features of genomes of eight PB-resistant K. pneumoniae isolates. Table S2. Allelic profiles of eight PB-resistant K. pneumoniae isolates. Figure S1. Paired SNP distance of eight PB-resistant K. pneumoniae isolates. Figure S2. PROVEAN result shows the effect of S85R in PmrB. [file 12866_2023_2808_MOESM1_ESM.docx]

**Table S1.** Features of genomes of eight PB-resistant *K. pneumoniae* isolates.

| **Genome** | **Genbank Accession** | **Genome Size (bp)** | **G+C (%)** | **Contigs (n)** | **CDS (n)** |
| --- | --- | --- | --- | --- | --- |
| KP14 | SAMN27478462 | 5,616,838 | 57.16 | 133 | 5646 |
| KP16 | SAMN27478463 | 5,538,404 | 57.49 | 107 | 5514 |
| KP17 | SAMN27478464 | 5,865,303 | 57.01 | 131 | 5948 |
| KP18 | SAMN27478465 | 5,685,521 | 57.18 | 127 | 5704 |
| KP20 | SAMN27478466 | 5,684,219 | 57.16 | 134 | 5701 |
| KP21 | SAMN27478467 | 5,866,112 | 57.01 | 133 | 5953 |
| KP24 | SAMN27478468 | 5,693,187 | 57.09 | 140 | 5746 |
| KP25 | SAMN27478469 | 5,863,695 | 57.01 | 129 | 5944 |

**Table S2.** Allelic profiles of eight PB-resistant *K. pneumoniae* isolates.

| **Strain** | **ST** | ***gapA*** | ***infB*** | ***mdh*** | ***pgi*** | ***phoE*** | ***rpoB*** | ***tonB*** |
| --- | --- | --- | --- | --- | --- | --- | --- | --- |
| KP14 | 11 | 3 | 3 | 1 | 1 | 1 | 1 | 4 |
| KP16 | 5254 | 3 | 3 | 1 | 1 | 515 | 1 | 4 |
| KP17 | 11 | 3 | 3 | 1 | 1 | 1 | 1 | 4 |
| KP18 | 11 | 3 | 3 | 1 | 1 | 1 | 1 | 4 |
| KP20 | 11 | 3 | 3 | 1 | 1 | 1 | 1 | 4 |
| KP21 | 11 | 3 | 3 | 1 | 1 | 1 | 1 | 4 |
| KP24 | 11 | 3 | 3 | 1 | 1 | 1 | 1 | 4 |
| KP25 | 11 | 3 | 3 | 1 | 1 | 1 | 1 | 4 |


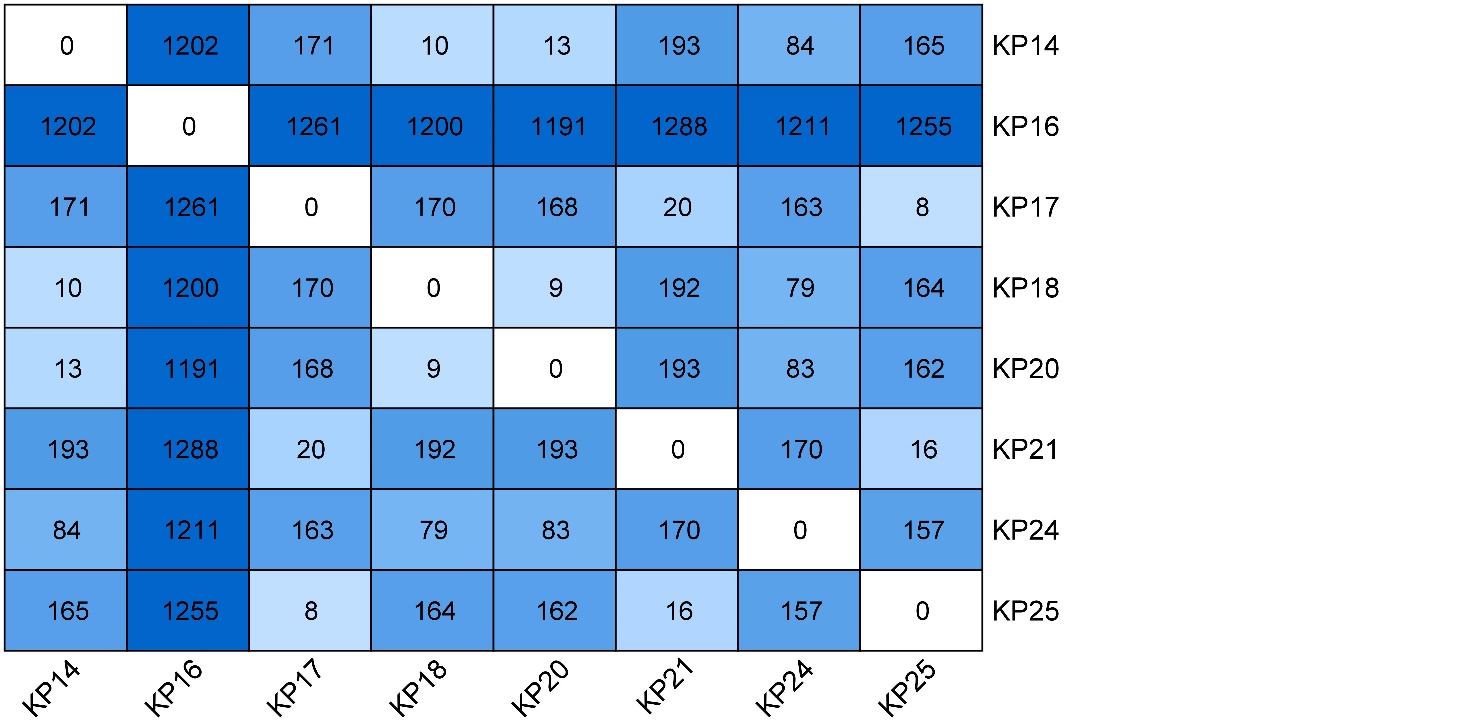
**Figure S1.** Paired SNP distance of eight PB-resistant *K. pneumoniae* isolates.


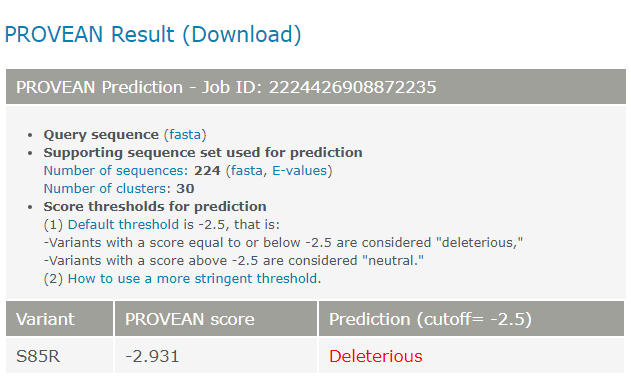


**Figure S2.** PROVEAN result shows the effect of S85R in PmrB.
